# Comparison of mortality in Asbest city and the Sverdlovsk region in the Russian Federation: 1997–2010

**DOI:** 10.1186/s12940-016-0125-0

**Published:** 2016-03-01

**Authors:** E. V. Kovalevskiy, S. J. Schonfeld, E. Feletto, M. Moissonnier, S. V. Kashanskiy, I. V. Bukhtiyarov, J. Schüz

**Affiliations:** Scientific Research Institute of Occupational Health of the Russian Academy of Medical Sciences, Moscow, Russian Federation; Section of Environment and Radiation, International Agency for Research on Cancer (IARC), 150 cours Albert Thomas, Lyon, 69008 France; Yekaterinburg Medical Research Center for Prophylaxis and Health Protection in Industrial Workers, Yekaterinburg, Russian Federation

**Keywords:** Russian Federation, Mortality, Neoplasms, Asbest, Sverdlovsk region

## Abstract

**Background:**

The Sverdlovsk region of the Russian Federation is characterised by its abundance of natural resources and industries. Located in this region, Asbest city is situated next to one of the largest open-pit chrysotile asbestos mines currently operational; many city residents are employed in activities related to mining and processing of chrysotile. We compared mortality rates from 1997 to 2010 in Asbest city to the remaining Sverdlovsk region, with additional analyses conducted for site-specific cancer mortality.

**Methods:**

Population and mortality data for Asbest city and Sverdlovsk region were used to estimate crude and age-specific rates by gender for the entire period and for each calendar year. Age-standardized mortality rates were also calculated for the adult population (20+) and Poisson regression was used to estimate standardized mortality ratios, overall and by gender.

**Results:**

During the period of 1997 to 2010, there were similar mortality rates overall in Asbest and the Sverdlovsk region. However, there were higher rates of cancer mortality (18 % males; 21 % females) and digestive diseases (21 % males; 40 % females) in Asbest and lower rates of unknown/ill-defined in Asbest (60 % males; 47 % females). Circulatory disease mortality was slightly lower in Asbest. Cancer mortality was higher for men in Asbest from oesophageal, urinary tract and lung cancers compared to the Sverdlovsk region. In women, cancer mortality was higher for women in Asbest from stomach, colon, lung and breast cancers compared to the Sverdlovsk region.

**Conclusions:**

This large population-based analysis indicates interesting differences but studies with individual exposure information are needed to understand the underlying factors.

**Electronic supplementary material:**

The online version of this article (doi:10.1186/s12940-016-0125-0) contains supplementary material, which is available to authorized users.

## Background

The Sverdlovsk region, an administrative area of the Russian Federation, is characterised by its abundance of natural resources and different types of industries. Many of these industries are situated in “monotowns” [1], a term referring to towns dominated by a single industry or company centred around these natural resources. Many monotowns in the Sverdlovsk region concentrate on the mining and processing of minerals. Several of those minerals are known to be carcinogenic to humans [2]. Asbest city is a monotown in the region situated next to one of the largest open-pit chrysotile asbestos mines currently operational. JSC “Uralasbest”, one of the main employers in the city, which produces approximately 20 % of the chrysotile used worldwide. Chrysotile asbestos, like all types of asbestos, is an established carcinogen [3–6]. For this reason, the Sverdlovsk region and Asbest city are compelling geographical areas in which to conduct detailed analyses of mortality, especially cancer.

Previous analyses based on data from 1997 to 2006 compared overall, and a limited number of cause-specific, mortality rates in Asbest city with those in the broader Sverdlovsk region [7]. Overall mortality was somewhat higher among females in Asbest than the region but the opposite was seen for males. For both males and females, mainly for older ages (≥59 years for males and ≥54 years for females), mortality rates were higher in Asbest city compared with the broader Sverdlovsk region for all cancers combined as well as for the groups of respiratory and digestive cancers [7]. Individual cancer sites were not investigated.

In this paper, we expand upon the above-referenced work and include data from 2007 to 2010, thus adding substantially to the number of deaths. The principal objectives were to describe and compare mortality rates in Asbest city with those of the rest of the Sverdlovsk region, with additional analyses conducted for site-specific cancer mortality.

## Method

Population and mortality data for Asbest city and Sverdlovsk region were obtained from the Sverdlovsk regional territorial body of the Federal State Statistic Service of the Russian Federation (FSSS). For purposes of comparing Asbest with the rest of the Sverdlovsk region, we subtracted the Asbest mortality and population data from the full Sverdlovsk region. Hereafter, the “Sverdlovsk region” refers to the region excluding Asbest city. The age distributions of the population (shown for three calendar years in Additional file 1: Figure S1) and total number of deaths (Additional file 2: Figure S2) were similar for Asbest city and the Sverdlovsk region. Mortality data were provided according to the Russian classification system of which different versions were used by the FSSS for 1997–1998 and 1999–2010, with a more detailed breakdown of causes of death provided after 1998. The classification systems were previously described in a study of Russian Federation mortality rates by cause and geographic region [8]. Briefly, the version used in 1997–1998 corresponds to the ninth revision of the International Classification of Diseases (ICD-9) [9] although some individual ICD-9 diagnoses are grouped together in the Russian system. The 1999–2010 version is more detailed and based on the ICD-10 [10]. Site-specific mortality rates for neoplasms were investigated where permitted by both coding systems. It was not within the scope of the study to access death certificates, which would have been useful in identifying cancer types that we problematic to isolate within the coding systems (e.g., mesothelioma).

Crude and age-specific rates per 100,000 persons were calculated by gender and region (Asbest city and Sverdlovsk region). Age-standardized mortality rates (ASRs) per 100,000 persons were estimated for the entire period (1997–2010) and for individual calendar years using the European age standard [11]. The data were restricted to ages ≥20, grouped into 5-year age groups and weighted as follows for calculation of ASRs: 20–24 (0.10), 25–29 (0.10), 30–34 (0.10), 35–39 (0.10), 40–44 (0.10), 45–49 (0.10), 50–54 (0.10), 55–59 (0.08), 60–64 (0.07), 65–69 (0.06), 70+ (0.10). Poisson regression models were used to estimate standardized mortality ratios (SMRs) and exact 95 % confidence intervals (95 % CIs) overall, by gender and by specific causes of death in Stata version 13.1, using the number of observed deaths in Asbest city as the outcome and the expected number of deaths (calculated by multiplying age-specific death rates in Sverdlovsk oblast (excluding Asbest city) to the Asbest city population) as the log offset. Confidence intervals excluding 1.0 were considered statistically significant. Heterogeneity between gender-specific SMRs was evaluated using likelihood ratio tests (LRT), comparing models with a single SMR estimate to those with gender-specific estimates with *P*-values < 0.05 considered statistically significant.

## Results

Between 1997 and 2010, there were a total of 16,596 deaths (52 % among males, 48 % among females) in Asbest town and 932, 699 deaths (53 % among males, 47 % among females) in the Sverdlovsk region at age ≥20 years. Table 1 shows the ASRs for the period of 1997 to 2010 for all major categories of mortality in Asbest City and the Sverdlovsk region as well as the SMRs comparing the two populations. Among males and females in both areas, the leading cause of death was from circulatory diseases. This was followed by deaths from cancer and external causes although the relative contribution of these two causes varied between groups; the mortality rate from cancer greatly exceeded that from external causes among females in both areas whereas in the Sverdlovsk region the mortality rate from external causes was markedly higher than that from cancer among males. Among males in Asbest city the mortality rates from cancer and external causes were similar. The fourth leading contributor to death was digestive diseases in all groups except for males in the Sverdlovsk region among whom respiratory deaths exceeded those from the digestive system diseases.

**Table 1:** Age-standardized rates (ASR) (per 100,000 person-years)^a^ and standardized mortality ratios (SMR) for major categories of death for ages 20+ in Asbest city and Sverdlovsk Region by Gender: 1997–2010

	Males				Females				Persons			
Cause of death	O _Asbest_^b^	ASR _Asbest_	ASR _Sverdlovsk_	SMR (95 % CI)	O _Asbest_^b^	ASR _Asbest_	ASR _Sverdlovsk_	SMR (95 % CI)	O _Asbest_^b^	ASR _Asbest_	ASR _Sverdlovsk_	SMR (95 % CI)
All-cause mortality	8661	2763.9	2801.7	0.98 (0.96–1.00)	7935	1388.5	1352.4	1.03 (1.00–1.05)	16596	1947.3	1941.6	1.00 (0.99–1.02)^c^
Infectious and parasitic diseases	220	61.9	68.2	0.90 (0.79–1.03)	65	14.6	14.5	1.04 (0.81–1.33)	285	36.1	38.9	0.93 (0.83–1.05)
Cancer	1503	496.6	416.2	1.18 (1.12–1.24)	1251	229.9	192.5	1.21 (1.14–1.28)	2754	322.3	270.5	1.20 (1.15–1.24)
Diseases of blood and blood forming organs	3	1.0	1.2	0.80 (0.17–2.34)	4	0.8	0.9	0.84 (0.23–2.16)	7	0.8	1.0	0.83 (0.33–1.70)
Endocrine, nutritional, metabolic diseases	34	10.7	7.5	1.41 (0.98–1.97)	74	13.0	9.9	1.38 (1.08–1.73)	108	12.5	9.2	1.39 (1.14–1.68)
Mental and behaviour disorders	16	4.3	8.2	0.51 (0.29–0.83)	8	1.6	2.2	0.75 (0.32–1.47)	24	3.0	5.1	0.58 (0.37–0.87)
Diseases of nervous system	52	15.7	20.1	0.76 (0.57–1.00)	57	10.8	9.3	1.26 (0.95–1.63)	109	13.2	14.0	0.96 (0.79–1.16)^c^
Diseases of circulatory system	4043	1354.6	1380.5	0.98 (0.95–1.01)	5059	837	862.7	0.97 (0.94–1.00)	9102	1042.2	1067.2	0.98 (0.96–1.00)
Diseases of respiratory system	359	114.3	162.3	0.71 (0.64–0.78)	207	37.6	36.8	1.04 (0.90–1.19)	566	66.9	83.8	0.80 (0.74–0.87)^c^
Diseases of digestive system	416	129.3	105.3	1.21 (1.10–1.33)	386	74.9	53.3	1.40 (1.28–1.55)	802	96.6	74.7	1.30 (1.21–1.39)^c^
Diseases of genitourinary system	54	17.7	16.0	1.09 (0.82–1.43)	77	14.6	9.5	1.55 (1.22–1.94)	131	15.5	11.7	1.33 (1.11–1.57)
All external causes^d^	1764	497.9	527.7	0.94 (0.90–0.99)	541	117.8	124.3	0.94 (0.87–1.03)	2305	290.4	306.4	0.95 (0.91–0.99)
Other causes of death^e^	44	14.7	13.1	1.11 (0.80–1.49)	161	27.5	17.4	1.61 (1.37–1.88)	205	23.5	16.2	1.46 (1.27–1.68)^c^
Ill-defined and unknown causes	153	45.1	75.3	0.60 (0.51–0.71)	45	8.5	18.9	0.47 (0.34–0.63)	198	24.3	42.9	0.57 (0.49–0.65)

^a^Age-standardized rates for ages 20+ calculated using European standard (5-year age groups 20–69, and 70+)

^b^Number of cases observed in Asbest city

^c^Male and female SMRs statistically significantly different (*p* < 0.05) based on likelihood ratio test

^d^For males and females combined, the largest ASRs for specific external causes were observed for homicides, suicides, other external causes, falls and motor vehicle accidents (traffic and non) in Asbest city and suicides, injury of undetermined intent, other external causes, unintentional poisoning by alcohol and homicides in Sverdlovsk region

^e^Other includes categories of pregnancy and childbirth, perinatal, congenital anomalies, benign tumours, otitis media and other diseases of mastoid processes, senility, diseases of skin, diseases of musculoskeletal system

For all persons (i.e., males and females combined), the mortality rates from cancer, endocrine, nutritional and metabolic diseases, digestive diseases, genitourinary diseases and other causes were significantly higher in Asbest than in Sverdlovsk region (SMRs 1.20 to 1.46) although only cancer and digestive disease contributed large numbers of deaths in either area. In contrast, mortality rates from respiratory diseases, mental and behavioural disorders, as well as external and ill-defined and unknown causes of death were significantly lower in Asbest City compared to the Sverdlovsk region (SMRs 0.57 to 0.95). Other causes of death for which SMRs were less than 1 but were not statistically significant include circulatory diseases, infectious and parasitic diseases, nervous system, and diseases of blood and blood forming organs. Statistically significant differences between male and female SMRs were observed for all-cause mortality as well as respiratory, nervous, digestive diseases and the category of “other” causes of death.

The mortality rate from all causes was slightly lower among males in Asbest City then in the Sverdlovsk region (SMR 0.98; 95 % 0.96–1.00) whereas it was marginally elevated for females (SMR 1.03; 95 % 1.00–1.05). For respiratory and nervous system mortality, the rates among males were significantly lower in Asbest than the Sverdlovsk region and this was not observed among females. Although mortality from digestive diseases was consistently higher in Asbest than the Sverdlovsk region, the SMR among females was greater than that for males.

**Fig 1:**
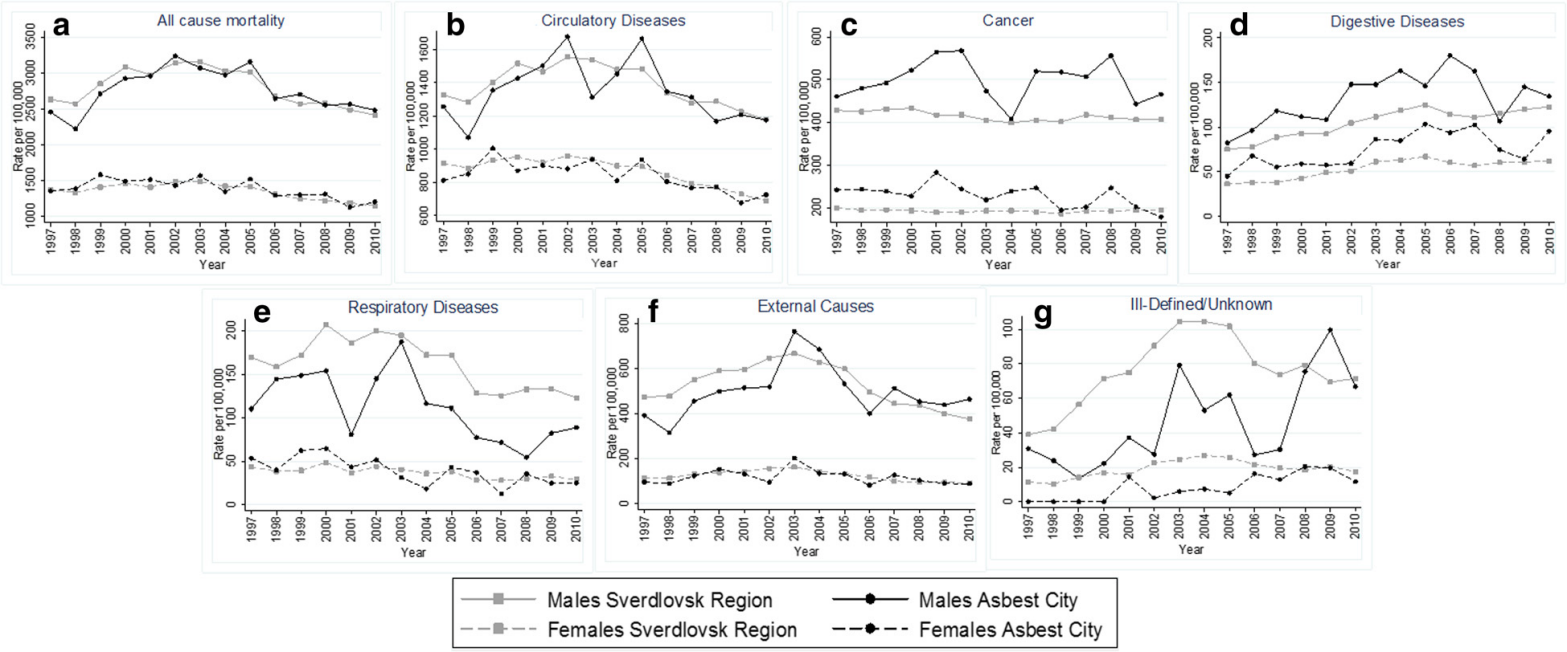
Age-standardized mortality rates among males and females 20+ years by calendar year (1997–2010) for select causes: **a**. All cause mortality **b**. Circulatory Diseases **c**. Cancer **d**. Digestive Diseases **e**. Respiratory Diseases **f**. External Causes **g**. Ill-Defined/Unknown

The yearly ASRs for all-cause mortality as well as for selected causes of death are shown by gender and region in Fig. 1. In both regions, mortality rates from these causes were consistently higher among males than females. Consistent with the smaller population size in Asbest city (Additional file 1: Figure S1), there was much greater fluctuation in the mortality rates over time in Asbest city than in Sverdlovsk region. These figures show a decline in the mortality rates from all-cause mortality as well as circulatory diseases, external causes and respiratory diseases among males and females in both Asbest city and Sverdlovsk region after 2005. In contrast, the rates of digestive disease mortality and mortality from ill-defined and unknown causes appeared to be increasing in all groups. These figures further illustrate that the pattern or direction of differences between Asbest city and Sverdlovsk region was generally consistent over time (i.e., consistently higher in Asbest than Sverdlovsk region or vice versa).

Table 2 shows the ASRs and SMRs from 1997 to 2010 for cancer mortality by site. In both regions, the leading causes of cancer mortality were lung cancer in males and breast cancer in females. This was followed by stomach cancer for both males and females. For all persons, the site-specific SMRs were significantly greater than 1 for cancers of the lung, oesophagus, stomach, colon, as well as for the groups of other digestive and urinary tract (SMRs 1.19 to 1.46). Breast cancer mortality was also significantly higher among women in Asbest city than the Sverdlovsk region. No significant differences between Asbest city and Sverdlovsk region were observed for mortality rates of other cancers. The only statistically significant difference between the male and female SMRs was for lung cancer [males - 1.20 (1.10–1.31); females – 1.76 (1.45–2.12)] but in both cases the rates were higher in Asbest city than Sverdlovsk region.

**Table 2:** Age-standardized rates (per 100,000 person-years)^a^ for cancer and standardized mortality ratios (SMRs), for ages 20+ in Asbest city and Sverdlovsk Region by Gender: 1997–2010

	Males				Females				Persons			
Cancer	O _Asbest_^b^	ASR _Asbest_	ASR _Sverdlovsk_	SMR (95 % CI)	O _Asbest_^b^	ASR _Asbest_	ASR _Sverdlovsk_	SMR (95 % CI)	O _Asbest_^b^	ASR _Asbest_	ASR _Sverdlovsk_	SMR (95 % CI)
Respiratory												
Larynx	48	15.6	12.0	1.29 (0.95–1.71)	3	0.7	0.4	1.40 (0.29–4.08)	51	6.1	4.7	1.30 (0.97–1.71)
Trachea, bronchus, lung	489	161.2	132.6	1.20 (1.10–1.31)	112	19.3	11.3	1.76 (1.45–2.12)	601	69.9	55.1	1.29 (1.18–1.39)^c^
Other respiratory	13	3.8	2.3	1.78 (0.95–3.05)	5	0.9	0.7	1.37 (0.44–3.20)	18	2.2	1.3	1.65 (0.98–2.61)
Digestive												
Oesophagus	51	16.9	11.3	1.46 (1.09–1.92)	15	2.6	1.8	1.43 (0.80–2.35)	66	7.6	5.3	1.46 (1.13–1.85)
Stomach	188	61.9	55.1	1.12 (0.96–1.29)	165	30.1	23.2	1.28 (1.10–1.50)	353	41.4	34.6	1.19 (1.07–1.32)
Small intestine	4	1.3	1.3	1.02 (0.28–2.61)	6	1.1	0.7	1.49 (0.55–3.24)	10	1.2	0.9	1.26 (0.60–2.31)
Colon	86	28.7	23.6	1.24 (0.99–1.53)	145	25.9	18.1	1.42 (1.20–1.67)	231	26.7	19.7	1.35 (1.18–1.53)
Rectum	80	27.9	22.5	1.20 (0.95–1.49)	73	12.7	12.6	1.04 (0.81–1.31)	153	17.5	15.8	1.12 (0.95–1.31)
Other digestive	145	47.2	36.5	1.29 (1.09–1.52)	126	22.6	20.5	1.09 (0.91–1.30)	271	31.7	26.6	1.19 (1.05–1.34)
Hematopoietic												
Leukaemia	33	11.4	9.1	1.18 (0.81–1.66)	37	7.1	5.1	1.37 (0.97–1.89)	70	8.2	6.5	1.28 (0.99–1.62)
Other hematopoietic	29	9.1	8.4	1.08 (0.72–1.55)	38	7.0	5.2	1.40 (0.99–1.93)	67	8.0	6.4	1.24 (0.96–1.58)
Male genital												
Prostate	67	23.4	21.5	1.09 (0.85–1.39)								
Other male genital	7	2.3	1.5	1.47 (0.59–3.03)								
Breast					194	37.8	32.1	1.19 (1.03–1.37)				
Female genital												
Cervix					35	7.0	8.3	0.86 (0.60–1.20)				
Uterus					51	9.7	9.0	1.05 (0.78–1.39)				
Other female genital					65	12.5	14.5	0.86 (0.66–1.10)				
Lip, oral cavity, pharynx	28	9.2	13.7	0.65 (0.43–0.94)	14	2.3	2.1	1.21 (0.66–2.03)	42	5.0	6.5	0.77 (0.56–1.04)
Urinary tract	116	38.8	29.4	1.31 (1.08–1.57)	40	7.1	6.8	1.05 (0.75–1.43)	156	18.2	14.7	1.24 (1.05–1.45)
Other/unspecified	118	37.7	35.1	1.07 (0.88–1.28)	127	23.4	20.0	1.20 (1.00–1.43)	245	29.0	25.6	1.14 (1.00–1.29)

^a^Age-standardized rates for ages 20+ calculated using European standard (5-year age groups 20–69, and 70+)

^b^Number of cases observed in Asbest city

^c^Male and female SMRs statistically significantly different (*p* < 0.05) based on likelihood ratio test

## Discussion

We compared mortality rates for the adult population of Asbest city, a monotown in the Sverdlovsk region developed around the mining and processing of chrysotile asbestos, with those for the rest of the region from 1997 to 2010. As observed for the region as a whole, the leading causes of death in Asbest city were from circulatory diseases, cancer, and external causes. This is consistent with previously published data from the Russian Federation [8, 12]. We also observed a decrease in all-cause mortality, circulatory diseases, external causes and respiratory diseases among males and females in both Asbest city and Sverdlovsk region after 2005; this recent decline in all-cause mortality and circulatory disease has been reported previously for other parts of the Russian Federation [13–15]. The mortality rates from cancer, endocrine, nutritional and metabolic diseases, digestive diseases, genitourinary diseases and the category of other causes were 20 % to 40 % higher in Asbest city than in the larger Sverdlovsk region whereas rates for respiratory diseases, mental and behavioral disorders, as well as external and ill-defined and unknown causes of death were significantly lower. The leading causes of cancer mortality were similar in the two areas (lung and stomach among men, stomach and breast among women) but for most sites examined, the rates were higher in Asbest city than the Sverdlovsk region.

One of the primary reasons for studying mortality patterns in Asbest is the city’s long history of chrysotile asbestos mining and processing activities. Asbestos, including chrysotile and all other forms, is an established human carcinogen [6]; it causes cancers of the lung, ovary, larynx as well as mesothelioma. Positive associations have also been reported for colorectal, stomach and pharyngeal cancers although the evidence is inconclusive [6]. Mortality rates for cancers of the lung, stomach, and colon were statistically significantly higher in Asbest city compared with the wider Sverdlovsk region. In a previous study of lung cancer incidence rates among males in Asbest city and the Sverdlovsk region found consistently elevated rates in Asbest city for the period of 1958 to 2008 [16]. Our findings are consistent with these results. In addition, there were non-significant elevated SMRs were also observed for cancers of larynx and rectum. Unlike lung cancer, non-malignant diseases of the respiratory system (composed largely of chronic lower respiratory diseases and pneumonia with few cases of conditions associated with external agents) were lower in Asbest city than in the Sverdlovsk region for males. We were not able to look specifically at pharynx or ovary cancers for the entire period due to the broad grouping in the cause-of-death classification. Furthermore, it was not possible to isolate mortality from mesothelioma as it is not coded separately in either version classification systems used by the FSSS, and there is no dedicated national register for the disease [17]. It has been shown in a review of mesothelioma cases reported from 1881 to 2006 in individual studies in the Russian Federation that over 80 % were of the pleura, with other types being very rare [17], which is similar to reported pleural mesothelioma cases in other countries [18]. As a result, any cases over the period included in our study would likely be included with “other respiratory cancers” which is elevated, but not significantly, in Asbest city compared to the Sverdlovsk region. However, there were only two known cases of malignant pleural mesothelioma in the Sverdlovsk region (personal communication with the Civil Statue Registry Office). Review of original text from death certificates, as would be obtained in studies based on individual rather than population data, would be needed to investigate this cause of death. As part of this register-based study, it was not possible to access the death certificates for other respiratory cancers to determine if they were due to mesothelioma. The increased mortality from several asbestos-related sites may be related to exposures among Asbest city residents, especially occupational exposure as many of the residents were employed in the mines, processing mills as well as manufacturing of other products from chrysotile, but there could be a complex influence of both occupational and environmental exposures present. Other explanations however cannot be dismissed, particularly as there was a general tendency towards higher rates in Asbest city than in the Sverdlovsk region for most cancer sites examined, including those not generally understood to be related to asbestos.

Mortality rates from external causes and digestive diseases, the third and fourth leading causes of death in Asbest city, also differed significantly from the regional rates. There were marginally lower mortality rates from external causes in Asbest city compared to the Sverdlovsk region whereas digestive disease mortality was elevated. Previous studies in the Russian Federation that have focused on mortality trends over time have highlighted the association between patterns of tobacco and alcohol consumption and multiple causes of death including cardiovascular and digestive diseases as well as external causes [8, 12, 14, 15, 19–21]. We observed somewhat lower mortality rates from circulatory disease and diseases of the respiratory system (non-malignant) in Asbest city compared to the Sverdlovsk region. However, in the absence of individual risk factor information, it is not possible to infer the extent to which differences observed between populations reflect differences in the distribution of risk factors and/or factors related to classification of cause of death. This is an inherent limitation of this type of ecological study.

Notably, we observed lower rates of mortality from ill-defined and unknown causes in Asbest city compared with the Sverdlovsk region although they were only 1 and 2 % of deaths respectively for persons age ≥20 years. Figure 1 showed an increase in the mortality rates due to unknown and ill-defined causes over time but the overall low prevalence of deaths that cannot be classified suggests a good quality of the data. The lack of individual information, discussed above, is a general limitation of studies based on population-level mortality data. Studies of this nature, however, benefit from the large number of cases from which to estimate mortality rates, although case numbers were still limited for less common causes of death including several individual cancer sites.

## Conclusion

In summary, this large population-based study indicates interesting differences between Asbest city and the rest of the Sverdlovsk region for some of the leading causes of mortality. Studies with individual exposure information are needed to understand the factors underlying these differences. One such study is currently underway to better understand the association between chrysotile exposure and the risk of cancer mortality among workers at the mine and processing mills of the JSC Uralasbest [22].

## Additional files

Additional file 1: Figure S1. Age distribution of males (green bars) and females (maroon bars) in Asbest City and Sverdlovsk Region in 1997, 2002, 2006. The different scales reflect the larger population of the Sverdlovsk region compared with Asbest city. (TIF 3500 kb) Additional file 2: Figure S2. Age distribution of deaths 1997–2010 for males (blue bars) and females (maroon bars) in Asbest city and Sverdlovsk region. The different scales reflect the larger population size and thus absolute number of deaths in Sverdlovsk region compared with Asbest city. (TIF 784 kb)
