# Supplementary figures and images for: Comparison of mortality in Asbest city and the Sverdlovsk region in the Russian Federation: 1997–2010
Source: Environ Health. 2016 Mar 1;15:42. doi: 10.1186/s12940-016-0125-0 (PMC4772512; doi:10.1186/s12940-016-0125-0)

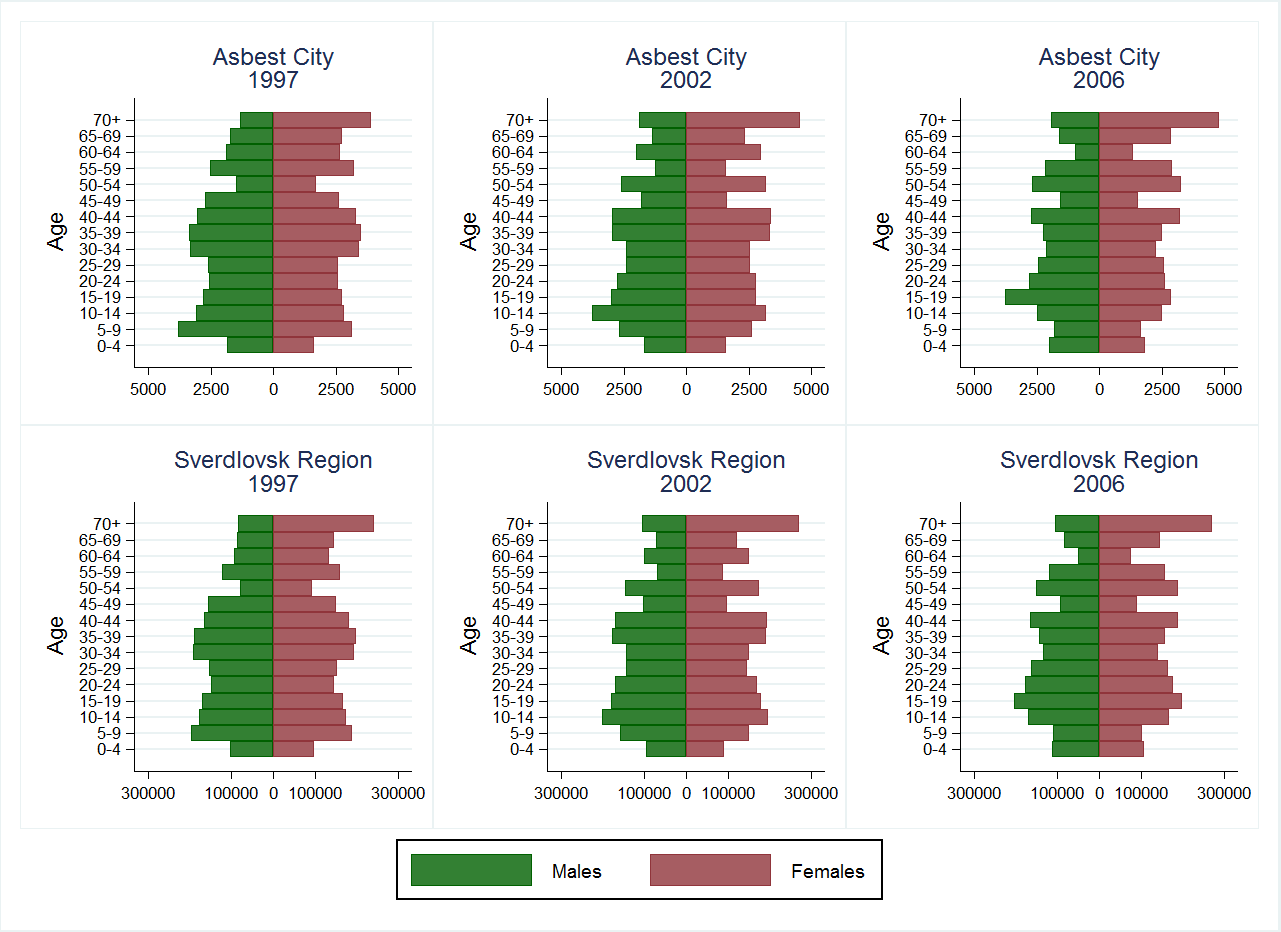

Supplement: Additional file 1: Figure S1. — Age distribution of males (green bars) and females (maroon bars) in Asbest City and Sverdlovsk Region in 1997, 2002, 2006. The different scales reflect the larger population of the Sverdlovsk region compared with Asbest city. (TIF 3500 kb) [file 12940_2016_125_MOESM1_ESM.tif]

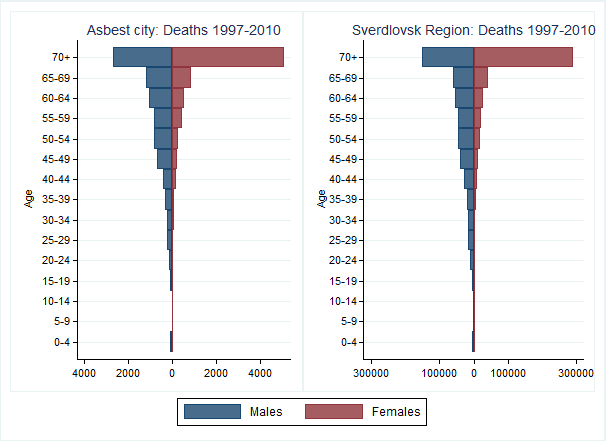

Supplement: Additional file 2: Figure S2. — Age distribution of deaths 1997–2010 for males (blue bars) and females (maroon bars) in Asbest city and Sverdlovsk region. The different scales reflect the larger population size and thus absolute number of deaths in Sverdlovsk region compared with Asbest city. (TIF 784 kb) [file 12940_2016_125_MOESM2_ESM.tif]
